# Supplementary material for: Modelling Skylarks (Alauda arvensis) to Predict Impacts of Changes in Land Management and Policy: Development and Testing of an Agent-Based Model
Source: PLoS One. 2013 Jun 6;8(6):e65803. doi: 10.1371/journal.pone.0065803 (PMC3675089; doi:10.1371/journal.pone.0065803)
Supplement: Supporting Information S4 — The skylark ODdox as a zipped archive. (ZIP) [file pone.0065803.s004.zip › Skylark_ODdox/globals_eval_0x73.html]

ALMaSS Skylark ODdox: File Members


ALMaSS Skylark ODdox
 2.0


- Main Page
- Related Pages
- Classes
- Files

- File List
- File Members

- All
- Functions
- Variables
- Typedefs
- Enumerations
- Enumerator
- Macros

- a
- b
- c
- d
- f
- g
- h
- i
- j
- l
- m
- p
- r
- s
- t
- w

### - s -

- sleep\_all\_day
  : treatment.h
- sob\_Clutch
  : skylarks\_all.h
- sob\_Female
  : skylarks\_all.h
- sob\_Male
  : skylarks\_all.h
- sob\_Nestling
  : skylarks\_all.h
- sob\_PreFledgeling
  : skylarks\_all.h
- sow
  : plants.h
- spring\_harrow
  : treatment.h
- spring\_plough
  : treatment.h
- spring\_roll
  : treatment.h
- spring\_sow
  : treatment.h
- start
  : treatment.h
- straw\_chopping
  : treatment.h
- strigling
  : treatment.h
- strigling\_sow
  : treatment.h
- stubble\_harrowing
  : treatment.h
- swathing
  : treatment.h
- syninsecticide\_treat
  : treatment.h


- Generated on Thu Jan 10 2013 13:15:39 for ALMaSS Skylark ODdox by
   1.8.1.1
